# Adolescents perception of reproductive health care services in Sri Lanka

**DOI:** 10.1186/1472-6963-8-98

**Published:** 2008-05-03

**Authors:** Suneth B Agampodi, Thilini C Agampodi, Piyaseeli UKD

**Affiliations:** 1 Medical officers of Health, Beruwala, Sri Lanka; 2 Director, National Institute of Health Sciences, Kalutara, Sri lanka

## Abstract

**Background:**

Adolescent health needs, behaviours and expectations are unique and routine health care services are not well geared to provide these services. The purpose of this study was to explore the perceived reproductive health problems, health seeking behaviors, knowledge about available services and barriers to reach services among a group of adolescents in Sri Lanka in order to improve reproductive health service delivery.

**Methods:**

This qualitative study was conducted in a semi urban setting in Sri Lanka. A convenient sample of 32 adolescents between 17–19 years of age participated in four focus group discussions. Participants were selected from four midwife areas. A pre-tested focus group guide was used for data collection. Male and female facilitators conducted discussions separately with young males and females. All tape-recorded data was fully transcribed and thematic analysis was done.

**Results:**

Psychological distresses due to various reasons and problems regarding menstrual cycle and masturbation were reported as the commonest health problems. Knowledge on existing services was very poor and boys were totally unaware of youth health services available through the public health system. On reproductive Health Matters, girls mainly sought help from friends whereas boys did not want to discuss their problems with anyone. Lack of availability of services was pointed out as the most important barrier in reaching the adolescent needs. Lack of access to reproductive health knowledge was an important reason for poor self-confidence among adolescents to discuss these matters. Lack of confidentiality, youth friendliness and accessibility of available services were other barriers discussed. Adolescents were happy to accept available services through public clinics and other health infrastructure for their services rather than other organizations. A demand was made for separate youth friendly services through medical practitioners.

**Conclusions and recommendations:**

Adolescent health services are inadequate and available services are not being delivered in an acceptable manner. Proper training of health care providers on youth friendly service provision is essential. A National level integrated health care program is needed for the adolescents.

## Background

Adolescence is stated as the period of transition from childhood to adulthood, which starts with the onset of puberty. It comprises the individuals between the ages of ten to nineteen years. During this important period, a child undergoes biological transition, which is characterized by puberty, related changes in physical appearance and the attainment of reproductive capability, psychological or cognitive transition, which reflects an individuals thinking, and social transition, which is related to rights, privileges and responsibilities of an individual[1].

In Sri Lanka, adolescents represent 22% of the total population[2]. School participation of the adolescents in 14–19 age group is around 55%[3]. The median age of marriage among Sri Lankan women has increased by almost seven years from 18.1 to 24.6 during the last century[2]. Data on unwanted pregnancies and abortions are not properly reported in Sri Lanka. However, studies have shown that young people in the 15–25 age group accounted for 19% of the illegal abortions that are taking place in the country[4,5]. The knowledge on available contraceptive methods are fairly high, and the use of contraceptive methods among married adolescents is around 65% [2]. Among unmarried adolescents the usage is not properly studied and there is a great paucity of data[6].

Premarital sex is not culturally accepted in Sri Lanka. But, in the country the sexual debut for both females and males is found to be around 15 years [7]. Prevalence of sexual activities (penetrative and non-penetrative sex) among school children is as high as 10.2% and among out of school adolescents it is 22.2%[7]. The 30000 estimated commercial sex workers [6] and the clearly reported low condom use [8] also increases the risk of transmission of HIV and STI. All these risk factors and low level of knowledge among adolescents, especially about transmission of HIV and STI [9] make young people more vulnerable for all kinds of Reproductive Health (RH) problems.

RH services in Sri Lanka are delivered as an integrated part of the family health program. The family health service delivery is conducted through the Medical Officer of Health (MOH) system where a community physician is in charge of a particular area. Average population covered by an MOH is around 60 000. An MOH area is further divided in to Public Health Midwife (PHM) areas where the average population is around 3000. PHM provides all family health services at the grassroots level, for her area.

The RH services in the family health program are traditionally targeted on married couples. However, RH services are available for those who seeks help through PHM, MOH, MOH clinics, youth corners in selected major hospitals and few Youth friendly Health Service (YFHS) centres. Availability of RH services is not adequate and even the places where such facilities are available are not accessible to those who really need the services[6]. Although some RH programmes targeted on the school children were carried out during the past few years, the most vulnerable population of recent school leavers is often being neglected[9].

Integrated services delivered through the healthcare system are identified as one of the most effective ways of delivering RH services [10]. This is a huge challenge in countries like Sri Lanka due to various cultural and social barriers. It is important that this service integration should be done in a very careful manner without disrupting the available system. In order to provide acceptable services with adequate utilization, in-depth exploration of social and cultural barriers and understanding the needs and expectations of adolescents is a great necessity.

This study intended to explore the perceived reproductive health problems, health seeking behaviors, knowledge about available services and barriers to access such services among adolescents in Sri Lanka. This would then help to develop and improve the delivery of existing adolescent RH services in a more efficient manner.

## Methods

### Study settings

Present study was conducted from March to May 2007 in the MOH area Beruwala, which is situated in the Kalutara district of Western province, Sri Lanka. The area is divided in to 47 PHM areas, each PHM area having around 3000 population each.

The area is vulnerable for RH problems, tourism being one of the major income sources in the area[11]. A considerable number of youth towards the coast tends to earn their living by serving as beach boys (Men having sex with men)[12]. Internally displaced people due to Tsunami and large-scale Tsunami constructions which causes internal migration of young workers to this area also contributes to the higher risk taking behaviours and vulnerability regarding RH issues (personal observations by authors as service providers). A YFHS project is currently being carried out in the area as a pilot project. Awareness raising and knowledge improvement was the main target in the first phase of the project. The next phase would be the integration of YFHS activities in to the present public health system in order to provide RH services in a more efficient manner.

### Study design

Authors used a qualitative method for understanding adolescents' perspectives on RH services. Traditionally used quantitative methods are unable to provide real life data on needs, believes, attitudes, and values of various population groups. Qualitative methods provides 'real life" rather than experimental or control views of past phenomena [13]. Authors used Focus Group Discussions (FGD) to achieve the study objectives. FGDs are widely used in exploring people's behaviours, perceptions, attitudes healthcare needs [14-18] and barriers to health services [18-20] in relation to RH.

### Participants

Adolescents aged 17 to 19 years and residing in the MOH area Beruwala participated in the study. The particular age group was selected as it covers both schooling and non-schooling populations. The school drop out rate in the area was highest after grade 10, which is around the age of 16 years.

### Sampling and recruitment of participants

Participants were selected from four PHM areas. Convenience sampling procedure was carried out to select these four PHM areas. Three PHM areas had local youth clubs, while the fourth had a local sports club. One research assistant contacted a member of each of these clubs and detailed him or her on the objectives of the research. The selected youth provided a list of eligible participants from the area. Eligible participants were those who belonged to the 17–19 year age group and have residing in the particular PHM area. The PHM and a research assistant visited these families, explained the nature of the study, and invited the subjects for the FGDs. All parents were provided with written information concerning the study and written consent was obtained. Each focus group had 6–11 Participants. Although most of the respondents were known to the PHM, there was no professional-client relationship between the investigators and the respondents prior to the study. Participants who were relatives of healthcare workers were excluded.

### Procedure

An interviewer guide was developed with the help of a Consultant Community Physician, a youth counsellor, a reproductive health physician and two Community physicians working in the study area. The Guide was developed in Sinhalese as the FGDs were conducted in the Sinhala language. Probing questions were also developed to explore RH problems. The guide consisted of open-ended questions related to broad subject areas of, perceived health problems, help seeking behaviour, knowledge on available RH services, barriers to reach such services and expectations of adolescents. Data collectors were trained according to the guidelines given by family health international on qualitative research[21]. The principal investigator (male) and a male research assistant conducted the FGDs for boys and a female investigator and a female research assistant conducted the FGDs for girls. The subjects and investigators were of the same sex to overcome the strong cultural barriers on discussion of reproductive and sexual health problems.

Discussions were carried out in places chosen by the participants. Each discussion lasted around one and half hours. At the end of the session, a summary of the recorded data was presented to participants and clarifications and corrections were made.

### Analysis of data

Tape-recorded data were fully transcribed and analysed manually. Thematic analysis was done to achieve study objectives. All transcripts were read several times by the investigators separately to bring out the main ideas, barriers and beliefs of participants. Then discussions among investigators were held to achieve common consensus on the most prevalent attitudes, barriers and beliefs expressed in each group. These themes were categorised according to perceived health problems, knowledge on available services, barriers to reach services, attitudes towards available services and expectations. Thematic analysis was performed and quotations were taken with common consensus of the group of investigators.

### Ethical and administrative considerations

Ethical clearance for the study was taken from the Ethical Review Committee, University of Colombo. Each participant and their parents were first provided with an explanation of the purpose, general content, and time commitment involved in participating in the discussion, and assurance of confidentiality. Informed written consent was obtained from each participant prior to discussions.

As the study involved discussion of sensitive issues, which could lead to further distress of the adolescents who are having problems, individual discussions and counselling services were offered to all participants after the discussions.

## Results

Altogether 32 adolescents participated in the discussions. The sample consisted of 13 males and 19 females. All of whom were between 17 to 19 years of age and had completed at least primary education. All were unmarried and 19 of them were still schooling. Three males were employed while 10 in the final sample had just finished school.

### Perceived health problems among adolescents

#### Common problems

Perceived problems among adolescent boys and girls differed. Both girls and boys reported that the most frequently encountered problems have lead to the development of psychological distresses among them. Love affaires, stresses due to exams, conflict of ideas with parents, lack of proper love and care by the family and lack of job opportunities were the main causes for psychological distress. Conflicts of ideas with parents were the main problem reported by the boys. Some explained that such conflicts were mainly due to the difference educational level between the younger generation and their parents.

"*Most of my friends are educated at least up to grade eight, while their parents had gone only up to grade five. So they can't understand what their children are doing*"

(18-year-old boy)

Both boys and girls discussed substance abuse as a prevailing problem among their friends. Some boys reported smoking and alcohol use as problems only among some groups. Furthermore, they also indicated that psychological disturbances due to masturbation, body shape and acne were also frequently discussed among their peers.

#### Reproductive health problems

Initially the girls were reluctant to reveal their RH problems. Eventually they came to a general agreement that problems regarding menstrual cycle were the commonest reproductive health problem among them. They also indicated that sexual harassment in many forms was another serious problem faced by them. Girls were agitated when they discussed this issue and the majority of them agreed that this had happened to them at least once, and mostly during public transportation (unwanted sexual touching) and in public places (verbal harassment) by unknown people.

Among the boys, the main RH problem discussed related to masturbation. Some forms of sexual harassment were also reported. However, boys faced these harassments usually from people known to them.

#### Adolescents' health seeking behaviours

In all FGDs, the adolescents agreed that friends were the first contact person for most of their health problems. Girls generally preferred advice from the mother especially for minor problems. Few girls indicated that they could discuss any matter with their mothers while the majority sought advice from their best friends. Only one participant reported that she could discuss reproductive health problems with a teacher. Nevertheless, most of the adolescent girls disagreed and said that they had no trust in teachers regarding these matters.

Boys agreed unanimously that they did not discuss these problems with parents or teachers. They described that, for minor problems they consulted their friends. However, for major problems they hesitated to consult even their friends.

According to the adolescent boys, marginalization and bulling among peer groups was very common, which was the reason why they did not want to be come out with such problems.

"*They may be friends, but you know we can't trust friends on this kind of matters, better to keep it to your self*"

(18-year-old young male)

"*You can forward the problem to your friends pretending that it has happened to one of your friends. Then they discuss it and you can get a clue to what to do*"

(17-year-young male)

Both boys and girls consistently described that parents always take them to private practitioners (allopathic) for RH problems. None of the subjects had experienced other types of therapies. The participants did not discuss the use of home remedies and traditional medicine.

#### Knowledge about available services

Knowledge on available services was very poor among adolescents. Only girls had heard about the availability of the PHM for their services at their doorstep. Not a single boy knew that they could seek help from the PHM or from MOH clinics for their RH problems. They were totally unaware of the availability of the youth corner in the nearby general hospital, and only two girls knew the availability of the youth friendly health services centre. None of them had visited this centre. Some boys complained that they are discriminated by the health care providers and policy makers.

"*Girls have various services and clinics at least twice a month in the village clinic centre. They also have clinics and specialists in hospitals for them. We boys do not have a single service, place or a person to discuss our problems*"

(17-year-old young male)

### Barriers to reach services

#### Lack of services

The main barrier for the adolescents was the unavailability of RH services. All participants agreed to this without any argument. They blamed health care providers, parents and all adults for neglecting them. Boys had more examples to report and they were frustrated about the lack of services.

"*No one cares about boys, but we have problems to discuss. We don't know whether these health care workers are good at solving our problems. The way they treat other illnesses made me feel uncomfortable to discuss sensitive reproductive issues with them*"

(17-year-young male)

"*They think we are healthy, but we have problems and we need answers*"

(19-year-young male)

#### Privacy and confidentiality concerns

Both boys and girls recognized lack of self-confidence and shyness as barriers in seeking help. Some of the adolescents explained that it was the main barrier for them to seek the services. Among girls, lack of confidence was accentuated due to the inadequate privacy and confidentiality given by health care providers, teachers and parents for these issues.

"*Doctor asked me embarrassing questions in front of my mother. As soon as we left the place she started asking me various questions with a tone of blaming; I decided not to seek medical advice again and not to tell anything to my mother*"

(18-year-old girl)

"*If you discuss these things, the principle and elder teachers will discriminate you in school*"

(17-year-old young female)

However, among boys, the impression of parents and teachers was not a problem but the major concern was about marginalization from their peer groups.

"*They will laugh at me if I seek medical advice for this kind of thing.*"

(17-year-old young male)

"*I can't go to my friends again if they get to know about this*"

(17-year-old young male)

#### Lack of knowledge and information

The groups pointed out how the lack of knowledge on RH among them had led to a lack of confidence in solving these problems. They described that information on reproductive and sexual health was deliberately made unavailable for them by their parents and their teachers. They thought that most of these obstacles would have been avoided if they were well aware of these issuse. They felt that these topics are usually kept away from them in schools and libraries. Even though they had some sources of information, they were not exposed to these at their correct age.

"*Two of my friends (both girls) ran away from home at the age of 14 and had a pregnancy with severe complications. They got to know about these things (even about sex) only after delivery of the baby. They did not have a clue about becoming pregnant with such an act. By that time even we didn't know exactly what was happening*"

(18-year-old young female)

Girls totally agreed with the above statement and most of them had similar stories to tell. They were in the opinion that RH education should start at least from age 13 onwards.

"*If you want to get the real picture, you go and have a discussion with children in grades 7 and 8, not with us*" was one comment received from an adolescent girl.

Attitudes of health care providers was another problem emphasized by the adolescents. They totally refused to go to crowded public clinics to discuss RH problems.

"*The doctor and the attendant are very busy to clear off the crowd. If you start long stories, they will ask you to cut it short. How can you cut it short when you do not know even how to start the story?*"

(19-year-old young female)

The adolescents were not happy even with present service of general practitioners. The majority of the participants were of opinion that doctors were not providing adequate information. Need for information was mentioned mainly in the male FGDs. The boys had numerous examples to elaborate their experiences.

"*When doctor treated me for mumps, I was so worried about impotence and infertility; that's what I heard from my friends, but he didn't spoke a word about complications*"

(18-year-old young male)

#### Attitudes of parents

Negative attitudes of parents, teachers and society were another barrier recognized by the adolescents, mostly by the girls. They reported that most of their mothers treated them as small kids. Some adolescent girls added that engaging in sex early in life was not a problem for some of the parents. Parents just tried to get rid of the girls by allowing them to practice sex and thereafter by allowing them to proceed with teenage marriages. They (adolescents) only realized that they had done a mistake when they reach their twenties. Most girls accepted this blame and they thought that the parents were responsible for problems related to early sex and marriages. However, boys had a rather different opinion and they said adolescents themselves were responsible for such acts.

#### Adolescents' expectations

All participants agreed that improving awareness on RH among their age group would be a main strategy in increasing service utilization by them. Girls suggested on improving awareness among teachers and parents as well. Furthermore, they appealed for continuous, effective advocacy programmes. However, boys stressed that they needed more knowledge for themselves. They had no claim in creating awareness among parents and teachers.

"*It is good that if someone can come to the house and speak on these things when parents and every body is there*"

(18-year-old young female)

"*Whether you educate them (parents) or not, we are not going to discuss these matters with them. Instead give us knowledge and provide us with services, so that we can manage these problems*"

(18 years old young male)

#### Need for well-trained health care providers

The adolescents emphasized that health personnel should be the ones to inform adolescents. Trained volunteers and other trained personnel (such as teachers) were not considered good enough. Service of the PHM was somewhat appreciated by girls and some of them were ready to contact the PHM for these services, if the PHM was willing to provide the services in a confidential manner. This would be a better choice as the PHM was already trusted and appreciated by their parents and therefore the girls could visit the PHM without any obstruction from their parents. However, they had some doubts regarding confidentiality.

"*She (PHM) is providing care only for pregnant mothers, so that, people will think that I'm pregnant and hiding something*"

(17-year-old young female)

Boys totally refused the service of midwives. They demanded for a young male doctor. Some of the girls also preferred a doctor.

"*If you tell the whole story to the PHM, she will refer you to a doctor. Then we should repeat the same embarrassing story once again. Why not have a direct contact with a doctor*"

(19-year-old young female)

In all FGDs the need for a doctor who could listen and understand their problems was mentioned. The girls liked to have an outsider to provide these services and some wanted a female doctor. However, some said that even male doctors would be very helpful.

"*We are used to male doctors in private places so that it doesn't matter as long as they are helpful*"

(18-year-old young female)

#### Need for special clinics

Use of existing clinics to provide adolescent health services was accepted by the majority of adolescent. Few girls explained that it was embarrassing for them to come to clinics, but others described that it was the ideal place. They said that those public clinics were safer and their parents' attitudes towards public clinics were favourable.

"*My mother will send me here but not to other places, especially to discuss reproductive health matters*"

(17-year-old young female)

Both boys and girls mentioned hat they needed special clinics in evenings or weekends. Most of them refused to come for services daytime (even for a separate session) when maternal and child clinics were conducted. The boys explained that they needed separate sessions without girls and they liked to have services on a different day.

## Discussion

This study provides an overview of adolescents' perceived reproductive health problems, health seeking behaviors, knowledge about available services and barriers to reach services in semi urban Sri Lankan context. Even though evidence is abundant through a decade of research on this subject [22], country and area specific problems should be investigated to provide services in a more acceptable manner. In Sri Lanka, few studies have attempted to explore qualitative aspects of adolescent health care needs and their perceived barriers [6].

Most of he current study findings are in parallel with other studies all over the world [22] But, some problems identified by the adolescents were unique to our study. Unlike previous findings, adolescents' major problem was psychological disturbances due to conflict with parents. Even health seeking behaviours were somewhat different from other settings. Evidence from developing and developed countries suggests that most of the adolescents seek help from friends and family[14]. However, this study sample was more internalized and, stigmatization from peers was one of their major concerns. Therefore, they were not willing to seek help from family and friends.

The negative attitude towards involving parents and teachers might be a sign of rapidly deteriorating family environment, especially for boys. This should be addressed immediately since parent child connectedness being one of the major determinants of adolescent health and risk taking behaviour [23-25].

Reported barriers to RH services was extensively discussed and highlighted repeatedly through the discussions. Availability, accessibility, acceptability, confidentiality and even lack of publicity and visibility of available services were the main barriers. These barriers have been recognized as universal barriers to adolescent health[26]. Need for reproductive health education was highlighted as a major step to remove these barriers. Although the adolescents in this study appealed for community based educational programmes through health care personnel, which was mainly due to the lack of confidence in teachers, evidence suggest that curriculum based sexual education show promising results on preventing adverse outcomes of adolescent RH problems [27,28]. So curriculum based programmes are needed, but it is also important to train teachers to tackle these sensitive issues in order to gain adolescents confidence.

Use of existing public health care infrastructure for adolescent health care services is a good strategy, considering the reported positive attitudes of adolescents. A recent review done by WHO has shown that this strategy was more successful than other strategies for RH service provision [29]. Acceptance of PHM as a service provider by adolescent girls is an encouraging sign. Improvement of confidential care and training in youth friendly service provision should, however, be encouraged. The 'family health worker" concept in Sri Lankan public health service, which is basically build upon maternal and child health care, can be widened by integrating RH concepts.

The biggest challenge for the effective implementation of programs would be the service provision for adolescent boys. With the context of family conflicts, lack of even peer group support, a huge gap in service provision and refusal of services available through the PHM, the situation is difficult. The only health care workers who could be utilized for his purpose would be the public health inspectors. However, this will be a major challenge for the public health service because at present the public health inspectors are not widely accepted in community as "family health workers". Adolescents' expectation of a trained medical practitioner as the primary health care provider is still not practicable. Initial contact point at grass root level should be through highly trained family health workers.

Services provided by youth friendly health service centre in the area are conceptually much in agreement with adolescents' needs. Nevertheless, there is a need to improve social marketing and accessibility of the services. According to the evidence available from previous studies[29] and attitudes of adolescents reported from this study, both sexes are not supportive of hospital-based services. The youth corner concept in hospitals should be re-evaluated and should be changed to provide more youth friendly services.

The present study opens a huge area for further qualitative research in order to gain more understanding of these problems. Parent-child-family conflicts and its impact on adolescent reproductive health is a priority area for studies.

The current study had several limitations. The number of FGDs conducted was limited and they were confined to the Sinhalese community only and the Muslim community was not involved. Involvement of parents, teachers and key informants (which was not done) would probably have given additional information in order to improve services.

In conclusion, according to adolescents' perception on availability, accessibility and acceptability of health services, present health care system has failed to provide appropriate services to them. Confidentiality and the quality of care is a major concern among adolescents. Planning of adolescents health care services should be initiated with participation of adolescents, so that the services will be more user friendly. Program planning should be based on qualitative studies in order to get a deeper understanding.

## Authors' contributions

SBA participated in the design, data collection, and manuscript preparation and performed the data analysis. TCA participated in the design, data collection and manuscript preparation. PUKD participated in design and helped to draft the manuscript.

All authors read and approved the final manuscript.

## Pre-publication history

The pre-publication history for this paper can be accessed here:


